# The Predictive Value of Impulsivity and Risk-Taking Measures for Substance Use in Substance Dependent Offenders

**DOI:** 10.3389/fnbeh.2019.00192

**Published:** 2019-09-19

**Authors:** Nathalie M. Rieser, Lilach Shaul, Matthijs Blankers, Maarten W. J. Koeter, Gerard M. Schippers, Anna E. Goudriaan

**Affiliations:** ^1^Department of Psychiatry, Amsterdam Institute for Addiction Research, Amsterdam UMC, University of Amsterdam, Amsterdam, Netherlands; ^2^Department of Research, Arkin Mental Health Care, Amsterdam, Netherlands; ^3^Netherlands Institute of Mental Health and Addiction, Trimbos Institute, Amsterdam, Netherlands

**Keywords:** addiction, dependence, criminality, violence, probation, BART, BIS/BAS, delay discounting

## Abstract

Impulsivity and risk-taking are known to have an important impact on problematic substance use and criminal behavior. This study examined the predictive value of baseline self-report and behavioral impulsivity and risk-taking measures [Delay Discounting Task (DDT), Balloon Analogue Risk Task (BART) and Behavioral Inhibition, Behavioral Activation Scale (BIS/BAS)] in 12-months follow-up substance use outcomes (e.g., use of alcohol, cannabis and other substances) and criminal recidivism (yes/no). Participants were 213 male offenders with a substance use disorder (SUD) under probation supervision. Bivariate regression analyses showed that BIS and BAS levels were associated (respectively) with the use of alcohol and cannabis. Multiple regression analysis showed that BIS was negatively associated with alcohol use at follow-up, whereas cannabis use at baseline and BAS predicted cannabis use at follow-up. At a trend level, interactions between delay discounting and risk-taking, and interactions between baseline cannabis use and BAS and BART predicted cannabis use at follow-up. Other substance use at follow-up was solely predicted by baseline other substance use. Overall, the findings provide marginal support for the predictive utility of impulsivity and risk-taking in accounting for variability in substance use among offenders with a SUD. This may be partly explained by the fact that only a limited number of psychological factors was assessed in this study. The studied population consists of a severe group, in which relapse into substance use or criminal behavior likely is related to complex, interacting biopsychosocial factors, of which impulsivity measures play a relatively small part.

## Introduction

This article examines the predictive utility of self-report and behavioral impulsivity and risk-taking measures on substance use in offenders with a substance use disorder (SUD). In SUDs, higher impulsivity has been linked to both the development of SUD, and to a more severe course, such as evidenced by earlier treatment dropout and more frequent relapses in SUDs (Stevens et al., 2014). Central to many dual-process theories about SUDs are the higher impulsivity and diminished control functions, compared with a focus on more immediate rewards, and specifically, responsivity towards drug-related cues, as for example in the I-RISA (Impaired-Response Inhibition Salience Attribution) model by Goldstein and Volkow (REF; Goldstein and Volkow, 2002; Verdejo-García and Bechara, 2009). This makes persons with SUDs who both experience a high reward responsivity to drug cues (e.g., by a higher cue reactivity, and a focus on more immediate rewards), in combination with less cognitive control—as for instance in higher impulsivity more vulnerable to relapse. A large number of studies corroborate that impulsivity and risk-taking are associated with a broad range of problematic behaviors such as SUDs or at-risk substance use (e.g., Moeller and Dougherty, 2001; Lejuez et al., 2003; Bornovalova et al., 2005; Perry and Carroll, 2008; Verdejo-García et al., 2008; de Wit, 2009; Dick et al., 2010; MacKillop et al., 2011; Smith et al., 2014) and criminal behavior (e.g., Gottfredson and Hirschi, 1990; White et al., 1994; Nofziger, 2009; Ribeaud and Eisner, 2016).

In addition, a strong and consistent association has been found between substance abuse and crime (e.g., Pihl and Peterson, 1995; Haggård-Grann et al., 2006; Bennett et al., 2008). Although impulsivity and risk-taking are associated with substance abuse and crime in general, these associations may differ across crime-types (e.g., violent, nonviolent; Cherek et al., 1997) and classes of substances (e.g., cocaine, heroin use; Bornovalova et al., 2005).

Impulsivity has been defined as “a predisposition toward rapid, unplanned reactions to internal or external stimuli without regard to the negative consequences of these reactions to the impulsive individual or to others” (Moeller et al., 2001, p.1784). It is a multifaceted construct whose facets can be assessed both by self-rated [e.g., Behavioral Inhibition, Behavioral Activation Scale (BIS/BAS)] and behavioral (neurocognitive) measures. Behavioral aspects of impulsivity are for instance delay discounting and risk-taking (Dougherty et al., 2015). The Delay Discounting Task (DDT) is a frequently used task that measures the preference for smaller immediate rewards over larger delayed rewards. The BIS/BAS scale is an instrument assessing reactivity to reward and punishment (Carver and White, 1994). Risk-taking behavior, the “propensity to seek out novel, stimulating but potentially harmful experiences” (Dougherty et al., 2015, p.1502) can be estimated with the Balloon Analogue Risk Task (BART), a computer task in which the propensity of an increase in gains (by pumping a balloon) over a risk of loss of the total accrued amount (when the balloon explodes after a pump) is measured.

Behavioral risk-taking as measured with the BART, and similar risky decision making tasks like the Cambridge Gamble Task—has been associated with substance use (Wills et al., 1994; Bornovalova et al., 2005; Schneider et al., 2012; Hanson et al., 2014). In this study, we, therefore, hypothesize that higher risk-taking would lead to a higher substance use, which was also shown by Fernie et al. (2010), who found that risk-taking predicted alcohol use in a group of 75 social drinkers. There are some indications that risk-taking in the BART is associated with alcohol use, but other studies indicate no differences. Ashenhurst et al. (2011) reported that higher risk-taking propensity was associated with lower alcohol use disorder symptoms in a sample of 158 non-treatment seeking heavy alcohol drinkers. Moreover, Ashenhurst et al. (2011) proposed that risk-taking may be an influential factor at initiation of alcohol use, but as use progresses, the relationship may turn in the opposite direction. Hanson et al. (2014) found a predictive effect of riskier choice in the BART and more frequent use of marijuana and other drug use in the past 18 months in a sample of 24 marijuana users and 34 non-users. Wichary et al. (2015) investigated risk-taking in male and female prisoners and non-prisoners. They reported an increased level of risk-taking in female prisoners compared to female non-prisoners, but no difference between male prisoners compared to male non-prisoners.

Delay discounting has also been associated with substance use, as indicated by two meta-analyses (Amlung et al., 2017). MacKillop et al. (2011) conducted a meta-analysis and reported a relation between substance use and delay discounting, but with a small magnitude of effect and high heterogeneity of effect size. They also presented a number of studies not showing a relationship for both alcohol and cannabis use. From the 64 studies analyzed, 27 studied substance use and 74% of the studies reported a higher delay discounting in the SUD samples compared to controls. In a more recent meta-analysis by Amlung et al. (2017), a small, but highly significant effect size was found for steeper delay discounting in SUD, and this relationship was stronger for studies focusing on severity of substance use problems, compared to studies including quantity by frequency measures of substance use. Results from single studies do indicate that several factors may impact the relation between substance use and delay discounting, as for instance gender: in a study on delay discounting and alcohol use in 65 college students or graduated students, higher levels of delay discounting were associated with higher levels of drinking in female college students, but not in their male counterparts (Yankelevitz et al., 2012). Although the meta-analyses did not indicate differential effects for specific substances, Moallem and Ray (2012) reported a steeper delay discounting rate in heavy drinkers who smoked (*n* = 213), compared to heavy drinkers (*n* = 107) or smokers (*n* = 67) solely. When drinking in combination with smoking is viewed as more severe substance use, this finding converges with the conclusion of the meta-analysis by Amlung, that severity measures have a stronger relation to steeper delay discounting. Impulsivity in substance using offenders was correlated with substance use in a sample of 80 drug court participants when measured using self-report, but not when impulsivity was measured with a DDT (Jones et al., 2015). In conclusion, both the meta-analyses indicate a link between substance use and delay discounting, whereas some other individual studies or in some subgroups—no relations were found. Thus, higher delay discounting in SUD samples is present, although the meta-analyses both indicate a small—but significant—magnitude of effect and high heterogeneity of effect size, indicating that the strength of the association of higher delay discounting in SUD samples differs across studies and may be stronger with more severe levels of substance use problems.

Regarding the relation between delay discounting and criminal activity, a smaller number of studies has been published. Åkerlund et al. (2016) analyzed the link between delay discounting at age 13 with their criminal behavior up to age 31 in 6,749 males. Adolescents showing a higher delay discounting rate had a higher risk to be involved in criminal behavior in the future. A study in 86 male offenders (prisoners and ex-prisoners) reported a significant group difference in delay discounting between non-offenders, prisoners and ex-prisoners; ex-prisoners showed a higher discounting rate compared to the other groups (Hanoch et al., 2013). Lastly, Piquero et al. (2018) recently conducted a long-term analysis in a longitudinal sample of over 400 boys of the predictive effect of delay discounting (instead of a task, they asked one question each at age 18, 32 and 48) on criminal behavior (number of convictions until age 56). Higher delay discounting was associated with more convictions.

Lee et al. (2017) found a bi-directional relation between delay discounting and property crime 1 and 2 years later in a study in 526 undergraduates. In another study among 63 male and female offenders, higher delay discounting rates were found in offenders compared to 70 non-offenders (Arantes et al., 2013). In the only study that examined offenders with a SUD, discounting rates and substance use among 80 offenders with a SUD were higher compared to noncriminal students (Jones et al., 2015). In a very small study by Cherek et al. (1997), parolees who had a history of violent crime (*n* = 9) displayed higher discounting rates than parolees without such a history (*n* = 21). Lastly, higher levels of delay discounting predicted property crimes, but not violent crimes later on Nagin and Pogarsky (2004). Mixed findings were present in a study by White et al. (1994), who reported a predictive value of cognitive and behavioral impulsivity at age 10 in a sample of 400 boys for delinquency at age 12–13, but no differences between stable non-delinquents, other delinquents and stable-serious delinquents in delay discounting at age 10 and 12–13. Wilson and Daly (2006) also found no difference in the discounting rates of young offenders (*n* = 91) compared to high school students (*n* = 284). Summarizing, previous research found evidence for an association between DDT and delinquency, but the relation has not uniformly been demonstrated, and studies in combined populations of offenders with problematic substance use are virtually non-existent (Jones et al., 2015).

BIS/BAS levels in young adults have been linked to alcohol, cannabis and methamphetamine use (e.g., O’Connor and Colder, 2005; Pardo et al., 2007; Simons et al., 2008). The BIS is associated with the avoidance of punishment, whereas the BAS is related to disinhibited behavior (Gray, 1975). The BAS was positively correlated with alcohol and cannabis use, whereas the BIS revealed a negative relation to these two substances (Pardo et al., 2007; Simons et al., 2008). To our best knowledge, there exists no previous research about the association of BIS/BAS levels with criminal recidivism and substance use in a criminal population. We hypothesize that higher BAS and lower BIS may promote substance use and criminal behavior.

In sum, relatively few studies assessed the association of impulsivity and risk-taking with substance use or future criminal behavior in offenders, using laboratory behavioral measures of impulsivity and risk-taking (e.g., Cherek et al., 1997; Mathias et al., 2002; Munro et al., 2007; Chen et al., 2008). These laboratory behavioral measures are especially important for the assessment of offender’s impulsiveness and risk-taking, as they provide measures that are less susceptible to simulation than self-report measures. In addition, very few of these studies have focused on multiple aspects of impulsivity. As impulsivity is a multifaceted construct, it can be argued that being impulsive on several of these aspects—e.g., having a focus on steeper delay discounting and a higher risk-taking propensity may exacerbate the effects on potential future substance use and criminal behavior more than only having a present reward orientation or high risk-taking. The purpose of our study is to examine the predictive utility of baseline self-reported and behavioral impulsivity and risk-taking measures, and interactions between impulsivity factors and baseline substance use and impulsivity measures on follow-up use of: (1) alcohol; (2) cannabis; (3) other substance use; and (4) criminal behavior in offenders with a SUD, using a self-rated measure of impulsivity (BIS/BAS) and behavioral measures of impulsivity (DDT) and risk-taking propensity (BART). We hypothesized that higher baseline scores on DDT, BAS and BART, and lower BIS scores would be associated with higher substance use (i.e., alcohol, cannabis and other substances) and higher criminal behavior at follow-up.

## Materials and Methods

### Study Design

A cluster-site, controlled trial (CRT) was conducted to examine the effectiveness of a brief motivation enhancing intervention for offenders with SUDs. The reported results were part of a larger study (see Shaul et al., 2016 for additional information). Within the 220 offenders under probation supervision, participants followed either the motivation enhancing sessions or supervision as usual. The probation officer was set as the cluster variable and the participants were allocated to the two conditions by cluster randomization. This means, 73 probation officers of six probation offices were randomized to perform either supervision with the motivation increasing intervention (intervention condition) or supervision as usual (control condition). With the allocation to the probation officer, participants were also allocated to the supervision they will follow. To control for a potential bias of the motivation enhancing intervention, only data of offenders from the control condition were included for substance use outcome.

### Recruitment and Assessment Procedures

The probation officers gave all the eligible offenders information about the study. The interested offenders were invited for the baseline assessment (T_1_). Baseline assessment took place in a private consulting room at the drug-probation office and consisted of a face-to-face interview, and three computerized neurobehavioral tests. A 17-inch laptop computer with a computer mouse was used to run the three neurobehavioral test-programs. Written informed consent for the offender’s study participation was obtained prior to baseline assessment. The follow-up (T_2_) took place on average 14.4 months (SD = 3.76) after baseline (T_1_). Offenders were paid €15 at baseline and €20 at follow-up for participation. The CRT was approved by the Medical Research Ethics Committee of the Academic Medical Centre, University of Amsterdam. The trial is registered at the Dutch Trial Register, number NTR2420.

### Participants

A total of 220 male parolees were included in the study, recruited from four addiction probation offices of five out of eleven District Courts in Netherlands (for more information regarding the inclusion process see Shaul et al., 2016). For 27 months, beginning in May 2010 until August 2012, all offenders meeting inclusion criteria were invited to participate. Inclusion criteria were: (i) a sufficient command of the Dutch language to understand interview questions and questionnaires; (ii) male gender; (iii) at least one prior sentence; (iv) regular use of alcohol and/or illicit drugs, i.e., using at least 3 days a week of which for alcohol: consuming at least five or more glasses per day; and (v) currently under a court-order supervision executed by an addiction probation service in a noncustodial setting. Exclusion criteria were: (i) a history of neurological problems or severe psychiatric disorders like schizophrenia, psychotic disorder, or bipolar disorder; (ii) only convicted for driving under influence; and (iii) illegal stay in the Netherlands. Of the 220 participants at baseline, 217 completed the DDT, 212 completed the BART and 209 filled in the BIS/BAS. We had to exclude two participants; one due to the diagnosis of schizophrenia (exclusion criterion i) and one participant due to not using any substances regularly (inclusion criterion iv). Five additional participants completed the BART or DDT at follow-up instead of baseline and were therefore excluded from our further analysis, leading to a final sample of *n* = 213. Out of the included 213 parolees, 160 participants finished the full procedure. Previous findings in our group showed that the motivation enhancing intervention had no significant effect on criminal recidivism at follow-up (Shaul et al., 2016), and effects of this intervention on treatment entry and substance use are being reported in a separate article (Shaul et al., 2016). Since the earlier publication found no difference between the two conditions regarding criminality, we used the whole sample (*n* = 213) to predict criminal behavior at follow-up. However, the effect of the motivation enhancing sessions on substance use at follow-up is still being analyzed, therefore, we used solely the participants in the no-intervention (*n* = 106) subgroup for the prediction of substance use at a 12-month follow-up.

### Measures

A semi-structured interview based on the MATE-crimi (Schippers et al., 2011) was conducted both at baseline (T_1_) and 12 months follow-up (T_2_) assessment, including demographic questions and questions regarding lifetime, 12 months and 30 days information from offenders about their substance use, treatment history and criminal behavior.

Substance use was measured both at T_1_ and T_2_ using the Measurements in the Addiction for Triage and Evaluation (MATE 2.1; Schippers et al., 2010, 2011). We distinguished between three classes of substances: alcohol, cannabis and other substances, and used different entities per class. For alcohol, we used the number of units of the last 30 days, for cannabis the amount of grams, and for other substances the total number of days used in the last 30 days before T_1_ and T_2_. Because use was lower for other substances (cocaine, crack, other stimulantia, ecstasy, heroine, other opiates and other substances), we added all these substances together and analyzed the effect of the aggregated use of other substances. As the measure of other substances variable included multiple substances it is possible that participants could show a sum of more than 30 days on T_1_ and T_2_ measures. This measure shows us how many days these substances were used.

#### Delay Discounting Task (DDT)

A computerized version of the DDT by Wittmann et al. (2007) was used to assess impulsive-choice behavior. This shorter functional magnetic resonance imaging (fMRI)-compatible version was included to limit the assessment time and in order to enable comparison to other SUD studies of the authors. The task consisted of six blocks, each containing eight trials on which participants made a choice between an immediate (lower) and a delayed (higher) hypothetical monetary reward. Delay in days (i.e., 5, 30, 180, 365, 1,095, 3,650) and delayed reward in euros (range 476–524 Euro) were equal for all trials of a given block, while the immediate reward value varied across trials within each block (range 0–476 Euro), in which the first two trials of one block were used to narrow down the delay equivalent depending on the responses made (see Wittmann et al., 2007 for exact adjustments). Block order varied and was randomized across participants. As proposed by Myerson et al. (2001), the area under the discounting curve (AUC) was used as a dependent measure; with lower AUC values denoting more discounting by delay (more impulsivity, or inversely, less self-control).

Data of 27 participants were considered non-systematic using the proposed algorithm developed by Johnson et al. (2010) to identify cases with indifference points that were not monotonically decreasing with delay. Specifically, a case was defined as non-systematic if: (i) two or more individual indifference points were greater than their preceding indifference point by a magnitude greater than 20% of the larger later reward; or (ii) the last indifference point was not less than the first indifference point by at least a magnitude equal to 10% of the larger later reward (Johnson et al., 2010). For participants with just one outlier point of indifference according to the former criteria (*N* = 11), the AUC was replaced by an adjusted AUC through linear interpolation of that point of indifference, leading to exclusion of 16 participants.

#### Balloon Analogue Risk Task (BART)

The BART (Lejuez et al., 2002) was used to assess risk-taking propensity. Due to time constraints, a version with 20 trials was chosen, as versions with 10–30 trials are a methodologically sound choice (Wallsten et al., 2005). As correlations for the total score are acceptable for the first 10 trials (~0.6) and good for trials 11–20 (~0.8) with little change for the 10 trials that follow (21–30: ~0.8; Wallsten et al., 2005; Dahne et al., 2013), we opted for a 20-trial BART version. This was also done for feasibility reasons (time restrictions). During each of the 20 trials, participants inflated a picture of a balloon by pressing a pump button on the screen with a laptop mouse. Each pump increased the risk of the balloon exploding (average breaking point being 64 pumps) and the potential earning (rising by 5 cents). In each trial the balloon’s potential earning that was accumulated in a temporary bank could be assured by clicking a collect button on the screen, thus transferring the earning from that particular balloon into a permanent bank. If a balloon exploded before that, the potential earning in the temporary bank for that balloon was lost and a new trial began. Participants received no precise information about the probability of explosion and the task contained no practice trials (for additional task details see Lejuez et al., 2002). The two outcome measures used were: (1) the total number of balloons that exploded during the task; and (2) the average number of pumps on trails where the balloon did not explode (i.e., adjusted average pumps).

#### Behavioral Inhibition, Behavioral Activation Scale (BIS/BAS Scale)

We used the BIS/BAS scale (Carver and White, 1994; Dutch version: Putman et al., 2004) to assess two general motivational systems underlying behavior. The BIS assesses the affective response to punishment, regulates avoidance of punishment and is associated with suppressing behavior and negative affect. The BAS assesses the affective response of upcoming rewards and is associated with the attainment of positively valued stimuli. The BAS scales are subdivided into the three categories; BAS drive, BAS fun-seeking and BAS reward sensitivity.

### Statistical Analysis

Statistical analyses were performed using SPSS 24.0 statistical software package (SPSS Inc., Chicago, IL, USA). Prior to the analyses, we converted all scores to z-scores entered into the bivariate, multiple, moderated and binary logistic regressions, in order for variables to have comparable impact. As suggested by Babyak (2004), we first conducted a bivariate linear regression with the potential predictors (DDT, BART, BIS/BAS) and the dependent variables (alcohol, cannabis and other substance use at follow-up) individually in order to minimize the amount of predictors* a priori* and prevent over-fitting of our models. The impulsivity and risk-taking measures with a *p*-value < 0.10 were included in the further analysis (Babyak, 2004). Second, we performed a multiple regression to investigate whether the measures examined* a priori* predicted the use of alcohol, cannabis or other substances in the last 30 days before 12 months follow-up assessment (T_2_). We used the exclusion criteria listwise as proposed by Field (2009). To correct for the baseline use of the specific substance (alcohol, cannabis, and other substances), we entered the T_1_ substance use measures in the first block. The impulsivity measures were entered in the second block, also using the enter method to assess which measures would show the highest impact. This resulted in three different multiple regression models for the prediction of alcohol use, cannabis use, other substance use at follow-up (T_2_). The impulsivity and risk-taking measures (BIS/BAS, DDT and BART) were entered using the forward step (likelihood ratio) method as suggested by Field (2009). The cut-off *p*-value to enter was set at 0.05 and the one to remove was set at 0.10 (Hosmer and Lemeshow, 2000).

Furthermore, to assess interaction effects of impulsivity measures on the multiple regression, we conducted moderated regressions. To predict substance use (alcohol or cannabis) at follow-up, we first entered substance use (either alcohol or cannabis use) at baseline and the hypothesized impulsivity measures into the first block and the interaction effect of the substance use at baseline with impulsivity measures and interactions between the impulsivity measures into the second block.

We hypothesize that a higher alcohol use at follow-up would be associated with a lower BIS, a lower delay discounting (present orientation) and their interactions (lower BIS in combination with lower delay discounting; lower delay discounting and higher BAS; lower BIS and higher BAS). We entered the measures as moderator, whereas alcohol use at baseline was set as the independent variable and alcohol use at follow-up as the dependent variable. In a second model predicting alcohol use at follow-up, we hypothesize that a lower BIS, a higher BART and interactions of the impulsivity measures (moderators) and of baseline alcohol use and impulsivity measures are associated with increased alcohol use. Analyzing the moderating effect of the impulsivity measures on cannabis use at follow-up—cannabis use at baseline as an independent variable—we expected a higher BAS and a higher delay discounting to be associated with higher cannabis use. Furthermore, higher values of combinations of impulsivity measures were expected to be linked to an increased cannabis use at follow-up.

## Results

Although not all variables were perfectly normally distributed, no serious violations of normality such as platy kurtosis requiring transformations were observed (Stevens, 1996). The demographic information is displayed in Table 1.

**Table 1:** Characteristics of offenders in the two groups.

	Samples	
	Substance use (*N* = 106)	Criminal recidivism (*N* = 215)
**Age mean (SD)**	37.55 (10.67)	37.03 (10.90)
**Years of education mean (SD)**	12.02 (2.28)	12.03 (2.31)
**Cultural identity % (n)**		
Dutch	57.5 (61)	57.8 (122)
Surinam/Antillean	24.5 (26)	23.7 (50)
Other	17 (17)	18.5 (39)
**Onset age criminal behavior mean (SD)**	19.67 (9.63)	20.15 (9.90)
**Onset age problematic substance use mean (SD)**	20.75 (7.96)	21.08 (8.68)
**Substance use at baseline in the last 30 days mean (SD):**		
Alcohol (units)	70.82 (133.81)	96.65 (233.32)
Cannabis (g)	21.90 (43.94)	28.65 (109.71)
Merged other substances (days)^a^	5.93 (13.87)	4.86 (13.06)
**Substance use at follow-up in the last 30 days mean (SD):**		
Alcohol (units)	88.66 (184.87)	101.40 (261.23)
Cannabis (gram)	14.51 (20.97)	14.31 (29.38)
Merged other substances (days)^a^	4.83 (12.83)	5.34 (14.56)
**Criminal recidivism at follow-up (yes) % (n)**	59.4 (63)	56.9 (124)
**BIS subscale (range: 7–28)**	17.25 (3.7)	17.9 (3.8)
**BAS subscale (score range: 16–52)**	39.5 (6.68)	39.8 (6.72)
**BART explosions (range: 0–14)**	4.8 (2.8)	4.7 (2.8)
**BART adjusted pumps (range: 1–64)**	28.5 (13.3)	28.5 (13.4)
**DDT AUC (range: 0.02–1.00)**	0.37 (0.28)	0.36 (0.26)

*Note: ^a^including: heroine, other opiate, crack, cocaine, other stimulantia, ecstasy and other substances*.

### Bivariate Analysis

When predicting alcohol use at follow-up in the bivariate analysis, only the BIS displayed a *p* < 0.10 and was therefore entered in the following multiple linear regression as only predictor. The same occurred for the BAS, which predicted cannabis use at follow-up and was included in the multiple regression for cannabis use. However, no impulsivity measure predicted the use of other substances with a *p* < 0.10 and no predictor was entered into the model. More detailed results of the bivariate analyses are shown in Table 2.

**Table 2:** Results of bivariate linear regression analysis for predictors of substance use individually.

Predictors	*β*	*R*	*R*^2^	*F*	*p*
**Dependent variable: alcohol use at follow-up**
BIS	−0.232	0.232	0.054	4.136 (1,73)	0.046*
BAS	0.138	0.138	0.019	1.415 (1,73)	0.238
DDT^a^	−0.179	0.179	0.032	2.115 (1,64)	0.151
BART^b^	0.112	0.112	0.012	0.936 (1,74)	0.336
**Dependent variable: cannabis use at follow-up**
BIS	−0.135	0.135	0.018	1.357 (1,73)	0.248
BAS	0.367	0.367	0.134	11.335 (1,73)	0.001*
DDT^a^	0.095	0.095	0.009	0.581 (1,64)	0.449
BART^b^	0.036	0.036	0.001	0.096 (1,74)	0.758
BIS	0.067	0.067	0.004	0.432 (1,97)	0.513
BAS	0.003	0.003	0.000	0.001 (1,97)	0.973
DDT^a^	0.084	0.084	0.007	0.625 (1,87)	0.431
BART^b^	0.056	0.056	0.003	0.313 (1,99)	0.577

Note: ^a^measured using Area under curve (AUC), ^b^measured using average adjusted pumps, *p < 0.10.

When predicting criminal recidivism for property crime, the BAS displayed a *p* < 0.10 and was therefore entered as the only impulsivity measure in the binary logistic regression analysis. No impulsivity measures showed a *p* < 0.10 when predicting criminal recidivism for violent crime or all types of crime. Detailed results of the bivariate analysis for criminal recidivism are reported in Table 3.

**Table 3:** Results of logistic regression analysis for predictors of criminal behavior individually.

	95% CI for odds ratio					
	B (SE)	Lower	Odds ratio	Upper	Nagelkerke *R*^2^	*p*-value
**Dependent variable: property crimes**
BIS	0.149 (0.152)	0.861	1.160	1.563	0.006	0.328
BAS	0.300 (0.161)	0.985	1.350	1.850	0.025	0.062*
DDT^a^	−0.018 (0.154)	0.726	0.982	1.329	>0.001	0.908
BART^b^	0.084 (0.147)	0.815	1.088	1.451	0.002	0.568
**Dependent variable: violent crimes**
BIS	0.072 (0.152)	0.798	1.075	1.449	0.002	0.634
BAS	0.245 (0.163)	0.929	1.278	1.758	0.016	0.131
DDT^a^	−0.204 (0.169)	0.585	1.137	0.816	0.011	0.229
BART^b^	0.014 (0.153)	0.751	1.014	1.368	0.000	0.929
**Dependent variable: all crimes together**
BIS	0.064 (0.140)	0.810	1.066	1.403	0.001	0.647
BAS	0.099 (0.138)	0.842	1.104	1.448	0.003	0.475
DDT^a^	−0.196 (0.146)	0.617	0.822	1.095	0.012	0.180
BART^b^	0.158 (0.141)	0.888	1.171	1.543	0.008	0.263

Note: ^a^measured using Area under curve (AUC), ^b^measured using average adjusted pumps, *p < 0.10.

### Multiple Linear Regression Predicting Substance Use in the Last 30 Days at Follow-Up

In the multiple regression analysis with alcohol use at follow-up as the dependent variable, the alcohol use at baseline was entered (*β* = 0.211, *p* = 0.073), the model was not significant; *F*_(1,71)_ = 3.310, *p* = 0.073, *R*^2^ = 0.211, adjusted *R*^2^ = 0.045. After including BIS (second block), the model showed a significant, albeit limited amount of explained variance; *F*_(2,70)_ = 3.770, *p* = 0.028, *R*^2^ = 0.312, adjusted *R*^2^ = 0.097, indicating a small goodness of fit according to Cohen, 1992). Alcohol use at baseline was marginally associated with alcohol use at follow-up (*β* = 0.217, *p* = 0.060), and the association with BIS was statistically significant (*β* = −0.230, *p* = 0.047).

In the first block of predicting cannabis use at follow-up, cannabis use at baseline entered the model with a *R*^2^ of 0.424 (adjusted *R*^2^ = 0.179), which indicated a small goodness of fit according to Cohen (1992). The model and the regression coefficient were significant; *F*_(1,73)_ = 15.96, *p* < 0.001, respectively *β* = 0.424, *p* < 0.001). Additionally in the second block, we found a significant prediction of cannabis use at follow up by cannabis use at baseline and BAS; *F*_(2,72)_ = 13.064, *p* < 0.001. The *R*^2^ for the overall model was 0.516 (adjusted *R*^2^ = 0.266), which also indicated a medium goodness of fit according to Cohen (1992). Therefore, the two variables together explained more variance than cannabis use at baseline solely. Cannabis use at baseline (*β* = 0.369, *p* = 0.001) and BAS (*β* = 0.300, *p* = 0.005) were significant predictors in the model.

### Moderated Regression Analyses

**Figure 1:**
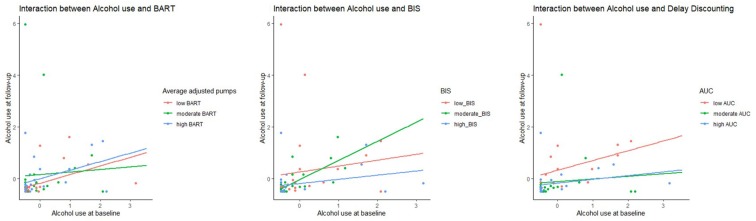
Scatterplot indicating the interaction between alcohol use and the measures Balloon Analogue Risk Task(BART, measured using average adjusted pumps), Behavioral Inhibition Scale (BIS) and Delay Discounting (measured using Area under curve, AUC). A higher measure on BART indicated a higher risk-taking because of more average adjusted pumps to a balloon. Whereas a lower AUC points to more discounting by delay, higher impulsivity and less self-control.

In the moderated regression analyses with alcohol use at follow up as the dependent variable—no predictors or interactions beyond the predictive value of the BIS (main effect of BIS (*β* = −0.28, *p* = 0.04), reached significance (*p* < 0.05) or a trend level (*p* < 0.10). The original model without the interactions, explained 18% of the variance (*R*^2^ = 0.18, adjusted *R*^2^ = 0.11); *F*_(5,61)_ = 2.5, *p* = 0.04. The moderated regression model did not reach a significant level (*p* = 0.18). In Figure 1, we report the interaction graphs between alcohol use and the measures BART, BIS and DDT.

**Figure 2:**
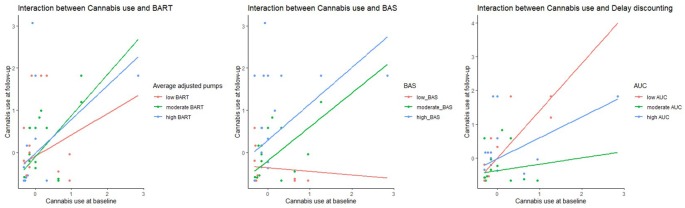
Scatterplot indicating the interaction between cannabis use and the measures BART (measured using average adjusted pumps), BIS and Delay Discounting (measured using Area under curve, AUC). A higher measure on BART indicated a higher risk-taking because of more average adjusted pumps to a balloon. Whereas a lower AUC points to more discounting by delay, higher impulsivity and less self-control.

**Figure 3:**
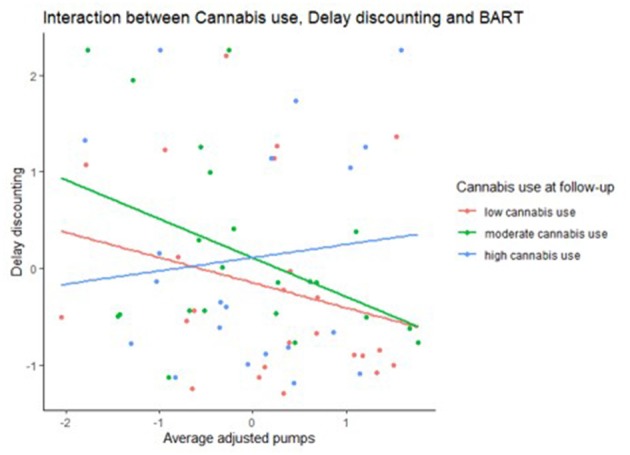
Scatterplot indicating the interaction between cannabis use and the measures BART (measured using average adjusted pumps) and Delay Discounting (measured usingArea under curve, AUC).

The moderated regression analysis to predict cannabis use at follow up, indicated in the first block, as expected from the multiple linear regression analyses, a significant main effect of cannabis use at baseline (*β* = 0.49, *p* ≤ 0.001) and BAS (*β* = 0.25, *p* = 0.02), but also of BIS (*β* = −0.22, *p* = 0.046). The moderated model also reached significance (*F*_(10,63)_ = 5.14, *p* ≤ 0.001, *R*^2^ = 0.49, adjusted *R*^2^ = 0.40). With the interaction effects in the model, besides the main effects of cannabis use at baseline (*β* = 0.32, *p* = 0.02) and BAS (*β* = 0.35, *p* ≤ 0.002), and a trend for BIS (*β* = −0.20, *p* ≤ 0.07), significant interactions were present for baseline cannabis use*BAS (*β* = 0.395, *p* = 0.013), a trend for baseline cannabis use*BART (*β* = −0.25, *p* = 0.09), and a trend for BART*delay discounting (*β* = −0.22, *p* = 0.046). To have a better understanding of the tendencies or direction, we included Figure 2 reporting the interactions between cannabis use and BART, BAS and DDT and in Figure 3 the interaction between cannabis use, BART and DDT.

To summarize, no significant interaction effects for prediction of alcohol use at follow-up were present, but in cannabis use at follow-up, interaction effects were present. In patients with a higher BAS, an increased use at baseline was strongly associated with higher cannabis use at follow up. However, in patients with a lower BAS, the association was minor. For the BART, lower levels of cannabis use at baseline in combination with higher risky decision making, predicted higher cannabis use at follow-up, whereas this relation was less strong in those with higher levels of cannabis use at baseline, although this only was a trend (*p* = 0.09). An interaction of both high BART and high delay discounting was predictive of more cannabis use at follow-up, although only at a trend level (*p* = 0.07).

## Discussion

We aimed to investigate the predictive value of impulsivity (DDT and BIS/BAS) and risk-taking (BART) measures for substance use and criminal recidivism at follow-up in a sample of substance using criminals. Results showed that self-rated impulsivity measures (BIS/BAS) were associated with substance use at follow-up. Specifically, a higher BIS predicted lower alcohol use at follow-up, whereas a higher cannabis use at baseline and BAS predicted an increased cannabis use at follow-up. For cannabis use, baseline use interacted with impulsivity measures to predict cannabis use at follow-up, and a (trend-level) interaction between delay discounting and risky decision making (BART) predicted higher cannabis use at follow-up. Other substance use at follow-up was not predicted by BIS/BAS impulsivity measures or any of the behavioral impulsivity measures and was only associated with baseline other substance use. Our hypotheses were therefore partly confirmed.

When looking at other substances, only baseline use was associated with use at follow-up. This may be due to a less frequent use of the other substances and a higher amount of non-users which resulted in a reduced power. The relationship between impulsivity, risk-taking and substance use might differ across substances in the other substance use class. For example, in a study in crack cocaine users, higher levels of risk-taking and impulsivity were present compared to those of heroin users (Bornovalova et al., 2005). Therefore, the combination of varying levels of impulsivity in the other substance use category may have had a reducing effect on power, although the analysis of the individual other substances was not an option, given their lower prevalence of use.

Previous research demonstrated that a higher BIS is associated with a more inhibited personality style (Hoppenbrouwers et al., 2015). Furthermore, higher BIS scores have been related to the avoidance of potentially dangerous environments (Campbell-Sills et al., 2004). Therefore, it seems plausible that increased inhibition, as evidenced in higher BIS scores are protecting for alcohol use as found in our study, which is also in line with previous findings in a study in undergraduates (Pardo et al., 2007). However, baseline alcohol use had no influence on alcohol use at follow-up, indicating that BIS had a stronger effect on future alcohol use than the baseline use, which means that personality traits relating to impulsivity may be more important when predicting future alcohol use.

Scores on the BAS have been associated with a higher attention to positively valued stimuli (Hoppenbrouwers et al., 2015), impulsive reward-seeking behavior (Carver and White, 1994) and disinhibited behavior (Gray, 1975). Therefore, a higher BAS could lead to a higher anticipation of pleasure and reward-seeking behavior, thus explaining the relation to increased cannabis use in our study in criminal offenders. This finding is in line with prior research (Pardo et al., 2007). Baseline cannabis use predicted follow-up use as well, and at a trend level, interactions between both higher risk-taking behavior as measured in the BART and higher delay discounting, and higher cannabis use at baseline and higher BAS were also predictive of higher cannabis use at follow-up, indicating that combinations of higher impulsivity have an additive effect on predicting cannabis use at follow-up, while also interacting with baseline cannabis use. In the last case, an opposite effect of the main effect of baseline cannabis use was present, with higher BAS and higher BART values impacting cannabis use at follow-up more when baseline cannabis use was lower.

No direct associations between BART or DDT and alcohol, cannabis, or other substance use were present in our study. This could be related to the fact that in this severe sample, probably with a higher level of impulsivity compared to the general population, the influence of these factors is limited through a restriction of range. Swogger et al. (2010) found no association between psychopathy and BART in male criminals and argued that caution is needed when generalizing results from non-criminal to criminal samples. They also suggested that the diagnostic benefit of the BART among inmates may be limited. To be able to detect differences between inmates and other samples, a BART version with higher rewards for risky behavior may be superior (Bornovalova et al., 2009).

As already seen in the introduction, some inconsistent results were reported in the literature regarding the association between DDT and offending, respectively. Prior research with the DDT demonstrated positive findings, for example, an increased delay discounting in offenders compared to students (Arantes et al., 2013), predictive value for property and violent crime in undergraduates (Lee et al., 2017), increased delay discounting in a drug court sample compared to non-criminal university students (Jones et al., 2015), and delayed discounting measured in early lifetime (13 or 18, 32 and 48, respectively) as a predictive value for criminal convictions until the age of 31 or 56, respectively (Åkerlund et al., 2016; Piquero et al., 2018). However, negative findings are also present for instance in two studies where: no differences in delay discounting between delinquents and non-delinquents were found (White et al., 1994; Wilson and Daly, 2006). Property and violent criminality may be associated differently with delay discounting, as high levels of delay discounting have been associated with property crime, but not violent crime, which was predicted by poor impulse control (Nagin and Pogarsky, 2004). Our results indicate no predictive value of DDT for criminal behavior, which may be related to the male sample that we included, whereas other studies included both males and females. Since gender differences exist in the DDT (Yankelevitz et al., 2012), this may have had an influence. The DDT used in this study was a shorter version than usual DDT tasks, which also may have led to a less optimal measurement of delay discounting, as also indicated by the exclusion of 16 participants due to non-systematic data on the DDT. Further, the statistical analyses differed; we wanted to predict criminal behavior and substance use in parolees, whereas the prior studies calculated a comparison between offenders and students or predicted crime in students or in a healthy sample. Thirdly, most studies analyzed a longer time period than we used (e.g., 18 years, Åkerlund et al., 2016; 38 years, Piquero et al., 2018). Lastly, the DDT itself differed; for example, Piquero et al. (2018) asked the participants only one question in each survey year to assess delay discounting, whereas Arantes et al. (2013) used a DDT with amounts between $500–$4,000 with delays between 1–8 years. These are higher amounts and later hypothetic payout than in the version of the DDT that was used in this study (Wittmann et al., 2007).

Another factor which may be related to future substance use that has been discussed in this field is one’s attitude towards the future. An optimistic attitude towards the future is linked to less risky behavior, whereas a negative orientation towards the future correlates with higher substance use and more risk-taking (e.g., Wills et al., 2001; Apostolidis et al., 2006a,b; Henson et al., 2006). Juveniles on probation who are more positive about their future were less involved in substance use and more likely to reject risky behaviors (Robbins and Bryan, 2004). The willingness to take risks can vary and be dependent on attitudes toward the future (Wilson and Daly, 2006). Participants in our sample recently left the prison and may show a more optimistic perspective for their future and may be keen to change their behavior. Therefore, they may display less impulsive behavior just after their stay in prison.

Wise and Koob (2014) discussed the development and maintenance of SUDs including positive (increase of behavior with positive stimuli) and negative (increase of behavior in order to remove or avoid a negative condition) reinforcement. When starting to use a substance, positive reinforcement, involving more impulsive behavior, is essential. They hypothesize that after developing an addiction, negative reinforcement predominates, involving elements of compulsivity [defined as “actions inappropriate to the situation that persist, have no obvious relationship to the overall goal, and often result in undesirable consequences” (Wise and Koob, 2014, p.257)]. Therefore, impulsivity may have a larger impact at the beginning of an addiction, but for the maintenance of an addiction, other factors may become more influential. Still, we found effects of the BIS/BAS, of a combination of high delay discounting and high BART and an interaction of BAS and BART with baseline cannabis use, on follow-up of cannabis use, indicating that combinations of higher levels of impulsivity and risk-taking can impact future substance (alcohol and cannabis use). These findings are consistent with addiction models that indicate a central role for impulsivity, other executive functions, and the underlying diminished functioning of the dorsolateral prefrontal cortex and anterior cingulate cortex (Goldstein and Volkow, 2002; Verdejo-García and Bechara, 2009). Thus, a combination of increased impulsivity, risk-taking and/or a preference for immediate rewards over delayed rewards, may exert its influence on future alcohol and cannabis, through changes in striatal-frontal brain circuitry, on top of the predictive effect that use at baseline has. Our sample consisted of parolees with a long history of criminal behavior and it may be possible that impulsivity at this period may have a smaller impact, than in younger or at-risk populations. This assumption may be supported by the findings in extremely violent prisoners, where Værøy et al. (2015) did not find an association between higher impulsivity (UPPS) and increased physical aggression (AQ-RSV). A further study found that premeditated aggression, which is defined as a planned action, predicted criminal recidivism, whereas impulsive aggression did not (Swogger et al., 2015), also indicating that the role of impulsivity may be limited, at least for some forms of (aggressive) criminality.

### Limitations

The sample consisted of male parolees and the effects are not generalizable to female offenders or offenders with comorbid mental illnesses. Additionally, the sample differed widely in the range of substance use. A few parolees had been using solely one substance, leading to a high portion of non-users in the “other substance” group (43.5%), limiting the power to detect differences for this specific analysis. Furthermore, the intervention or probationary service which all of these parolees followed, could have had a diminishing effect on impulsivity, meaning that the sessions may have reduced the influence of impulsivity on substance use. In this study, we could explain between 10%–40% of variance, which means that there are other predictors that we did not measure, such as other personality traits, acute substance intoxication during offense, childhood experiences, genetic predisposition, their neighborhood and its criminogenic behavior and/or relationships (Zimmerman, 2010). In this study, no counterbalancing was employed and thus, fatigue may have impacted the neurocognitive assessment, potentially impacting the power of predicting substance use at a later point. For the delay discounting, a quality check was done ensuring data integrity (see DDT), indicating that only in a small minority of cases, this was the case. Also, given the fact that the DDT and BART were not speed tasks, we think fatigue only may have impacted the data minimally. Another limitation may lie in a “restriction of range” effect: as our sample consisted of criminals with problematic substance use, this likely reflects a population in which impulsivity is higher than in the general population, and thus, impulsivity measures may have had more limited effects in our study. Lastly, the analyzed time at risk may have been too short. Further studies should assess a longer time period after prison.

## Conclusion

Several impulsivity and risk-taking tasks had a predictive impact on alcohol and cannabis use at follow-up in male, substance using parolees. Assessing behavioral inhibition, behavioral activation, impulsivity and risk-taking propensity in parolees seem to be a valuable addition in order to prevent substance use. Using these scores, parolees could be assigned to an intervention that focuses on reducing impulsivity and/or risk-taking behavior. However, additional research is needed in order to improve the assessment of predicting criminal recidivism and substance use, taking into consideration other variables which may explain the complex roles of impulsivity and risk-taking in criminal behavior and substance use.

## Ethics Statement

The trial was approved by the Medical Research Ethics Committee of the Academic Medical Centre, University of Amsterdam. The trial is registered at the Dutch Trial Register, number NTR2420. Participants provided written informed consent prior to study participation.

## Author Contributions

GS, MK, and LS designed the study. NR and LS drafted the first version of the manuscript. AG and MB amended the draft and critically reviewed the article. All authors approve submission of the manuscript.

## Conflict of Interest Statement

The authors declare that the research was conducted in the absence of any commercial or financial relationships that could be construed as a potential conflict of interest.
